# Plausible diagnostic value of urinary isomeric dimethylarginine ratio for diabetic nephropathy

**DOI:** 10.1038/s41598-020-59897-1

**Published:** 2020-02-19

**Authors:** Dharmeshkumar Parmar, Nivedita Bhattacharya, Shanthini Kannan, Sangeetha Vadivel, Gautam Kumar Pandey, Avinash Ghanate, Nagarjuna Chary Ragi, Paramasivam Prabu, Thyparambil Aravindakshan Pramodkumar, Nagaraj Manickam, Viswanathan Mohan, Prabhakar Sripadi, Gokulakrishnan Kuppan, Venkateswarlu Panchagnula

**Affiliations:** 1 Biochemical Engineering, CSIR-National Chemical Laboratory, Dr. Homi Bhabha Road, Pune, 411008 India; 2 Department of Research Biochemistry, Madras Diabetes Research Foundation, No. 4, Conran Smith Road, Gopalapuram, Chennai 600086 India; 3 Department of Vascular Biology, Madras Diabetes Research Foundation, No. 4, Conran Smith Road, Gopalapuram, Chennai 600086 India; 4 Academy of Scientific and Innovative Research, CSIR-NCL Campus, Dr. Homi Bhabha Road, Pune, 411008 India; 5 CSIR-Indian Institute of Chemical Technology, Uppal Road, Hyderabad, 500007 India; 6 Department of Neurochemistry, National Institute of Mental Health and Neurosciences, Hosur Road, Bengaluru, 560029 India

**Keywords:** Predictive markers, Diabetes complications

## Abstract

Altered circulatory asymmetric and symmetric dimethylarginines have been independently reported in patients with end-stage renal failure suggesting their potential role as mediators and early biomarkers of nephropathy. These alterations can also be reflected in urine. Herein, we aimed to evaluate urinary asymmetric to symmetric dimethylarginine ratio (ASR) for early prediction of diabetic nephropathy (DN). In this cross-sectional study, individuals with impaired glucose tolerance (IGT), newly diagnosed diabetes (NDD), diabetic microalbuminuria (MIC), macroalbuminuria (MAC), and normal glucose tolerance (NGT) were recruited from Dr. Mohans’ Diabetes Specialties centre, India. Urinary ASR was measured using a validated high-throughput MALDI-MS/MS method. Significantly lower ASR was observed in MIC (0.909) and MAC (0.741) in comparison to the NGT and NDD groups. On regression models, ASR was associated with MIC [OR: 0.256; 95% CI: 0.158–0.491] and MAC [OR 0.146; 95% CI: 0.071–0.292] controlled for all the available confounding factors. ROC analysis revealed ASR cut-point of 0.95 had C-statistic of 0.691 (95% CI: 0.627-0.755) to discriminate MIC from NDD with 72% sensitivity. Whereas, an ASR cut-point of 0.82 had C-statistic of 0.846 (95% CI: 0.800 - 0.893) had 91% sensitivity for identifying MAC. Our results suggest ASR as a potential early diagnostic biomarker for DN among the Asian Indians.

## Introduction

Diabetic nephropathy (DN) is among the most significant longer-term complications associated with diabetes mellitus. The risk of end-stage renal disease (ESRD), resulting premature morbidity and mortality are estimated to increase with diabetes by 12 fold^1^. A recent epidemiological study reported an increase in mortality rates associated with renal complications in diabetes^2^. Microalbuminuria (MIC) is an early non-invasive marker of renal disease and its progression. However, it takes several years of diabetes for MIC to occur. Interventions are also much less effective in some patients with MIC who manifest advanced pathological changes^3^. Development of sensitive and early stage markers along with alternative diagnostic approaches are thus essential for the detection of DN.

Symmetric and asymmetric dimethylarginines (SDMA and ADMA) are structural isomers. They are formed in the protein methylation biosynthetic pathway when methylated protein arginine residues are hydrolyzed and released^4^. These isomers were originally discovered from urine in a relatively high abundance^5,6^. ADMA is predominantly metabolized to citrulline and dimethylamine by dimethylarginine dimethylaminohydrolase (DDAH1)^7^. About 10% of ADMA, however, is eliminated via urinary excretion. The disruption of the DDAH pathway leading to the build up of ADMA has been implicated in its role as an NOS inhibitor^8,9^. Overexpression of renal DDAH and increased urinary elimination of ADMA reduces renal injury in DN^10^. Separately, SDMA could induce arginine deficiency in the endothelial cells thereby reducing the production of nitric oxide (NO)^11^. Increased risk of chronic kidney disease (CKD) progression and atherosclerotic cardiovascular events have been shown to be associated with elevated ADMA and SDMA levels^12,13^. Our earlier studies showed that circulatory ADMA had a causative role in the development of DN^14^.

ADMA to SDMA ratio (ASR), also known as catabolism index, has been implicated in sepsis, hypertension, malnutrition, and inflammation^15–19^. A direct correlation between malnutrition, inflammatory markers, and ASR was also established in patients undergoing hemodialysis^16^. The inverse of ASR (SDMA/ADMA ratio) has been reported to correlate with an atypical renal function that can possibly delineate renal insufficient subjects from healthy controls^20^. Altered individual ADMA and SDMA levels were found in patients with end-stage renal failure^21–24^. Hence, we hypothesize that altered urinary ASR could also be potential early biomarker for DN.

Asian Indians are known to be more prone to insulin resistance^25^, predisposed to cardio-metabolic diseases, and DN^26–28^. However, there is a lack of data on ASR in relation to nephropathy in this high-risk population. This investigation evaluates the efficiency of ASR as a potential marker for early diagnosis of nephropathy in Asian Indians with varying levels of glucose intolerance as well as in patients with T2DM with or without DN.

## Research Design and Methods

Participants for the study were recruited from Dr. Mohans’ Diabetes Specialties Centre, a tertiary diabetes care centre in Chennai, India. In this cross-sectional study, individuals with (a) normal glucose tolerance (NGT; *n* = 95), (b) impaired glucose tolerance (IGT; *n* = 80), (c) newly diagnosed diabetes (NDD; *n* = 120), (d) T2DM with microalbuminuria (MIC; *n* = 140), and (e) T2DM with macroalbuminuria (MAC; *n* = 120) were recruited. This study being cross-sectional in nature, no cause and effect relationship or mechanistic conclusions between ASR and NDD, MIC or MAC could be established. Institutional Ethics Committee (IEC) approval (from the Madras Diabetes Research Foundation, Chennai and National Chemical Laboratory, Pune) was obtained prior to the start of the study; all research methods were performed in accordance with relevant guidelines/regulations and written informed consent was obtained from all the study participants.

### Sample preparation

Urine samples were collected and immediately stored at −80 °C in 100 µL aliquots until further processing. To begin sample processing, the samples were thawed on ice. 400 µL cold methanol was added to an aliquot and the content was thoroughly vortexed. Subsequently, the samples were subjected to centrifugation at 13,200 rpm, 4 °C for 15 minutes before collecting ~250 µL of the supernatant for MALDI MS/MS analysis. The sample processing method was uniformly used for the samples, quality controls, and pooled samples. Pooled samples were used for method standardization, performance evaluation. A subset of samples was used to perform cross-platform validation by LC-MS/MS^29,30^.

Urinary matrix effects were evaluated using pooled samples spiked with albumin. 100 µL of 5 randomly chosen urine samples were mixed to prepare two pooled urine samples, one each for NGT and MAC category. Effects of the presence of protein on the ASR measurement was evaluated by – pooled NGT spiked with five levels of BSA simulating the conditions of MIC, MAC, and two very high concentrations of BSA. The ASR values in spiked samples were compared against NGT pooled without the protein spike **(**Supplemental Table 1).

### Mass spectrometric estimation of ASR

We previously reported a matrix-assisted laser desorption/ionization mass spectrometry (MALDI MS) absolute quantification method and demonstrated it for the estimation of ADMA and SDMA concentrations from urine^31^. For this study, an optimized and validated MALDI MS/MS method^32^ has been employed to selectively measure ASR using the signature product ion ratio of ADMA and SDMA. The method was thoroughly tested for precision, accuracy, performance, and cross-platform validation (please see Supplemental Fig. 1 to 7 and Supplemental Table 1 to 4 for full details of method standardization and validation).

MALDI-MS/MS was performed on AB Sciex 5800 MALDI TOF/TOF mass spectrometer with collision-induced dissociation (CID) cell. The instrument is equipped with Nd-YAG laser operating at 345 nm, pulse length - 500 ps and repetition rate - 1000 Hz. CID was performed at 1 kV in positive ion mode and the pressure was maintained at 1 × 10e-6 Torr. Optimized laser fluence at 5200 units, delayed extraction (DE) time at 200 ns, 2000 laser shots and a precursor selection window 203.15 ± 0.5 *m/z* was used for MS/MS analysis. Detector voltage was optimized at 2.69 eV for small molecule analysis. Representative MALDI-MS/MS spectra are showcased in the Supplemental File **(**Supplemental Fig. 1**)**.

To perform MALDI-MS/MS analysis, the 0.5 µL of supernatant described above was applied on 96-well MALDI target plate pre-spotted with CHCA matrix (10 mg/mL). Subsequently, the 96-well MALDI target plate was dried overnight before analysis. The data was acquired in an automated fashion with a randomized laser fire pattern.

Data analysis was performed using an in house developed data processing tool “MQ” (http://www.ldi-ms.com/services/software). The raw instrumental files (T2D) were converted into a generic ASCII format using a T2D converter (software version 1.0) prior to processing using ‘MQ’. ADMA and SDMA peak intensities were estimated using the characteristic product ions of ADMA and SDMA at *m/z* 46 and *m/z* 172, respectively. The observed product ions *m/z* in MS/MS that selectively confirm the presence of the respective isomers were in agreement with previously reported results^31^. The mass extraction window (MEW) was set to 50 ppm. ASR was estimated as an accurate mass extracted peak-intensity ratio of the two diagnostic ions (at *m/z* ~46 and 172 for ADMA and SDMA respectively). The MALDI MS/MS method was optimized to be free of interferences associated with ion suppression or sample matrix. The method was also validated using LC-MS/MS on a subset of samples **(**Supplemental Figs. 3, 4 and Table 3**)**. Details are available in the Supplemental File.

## Results

**Figure 1:**
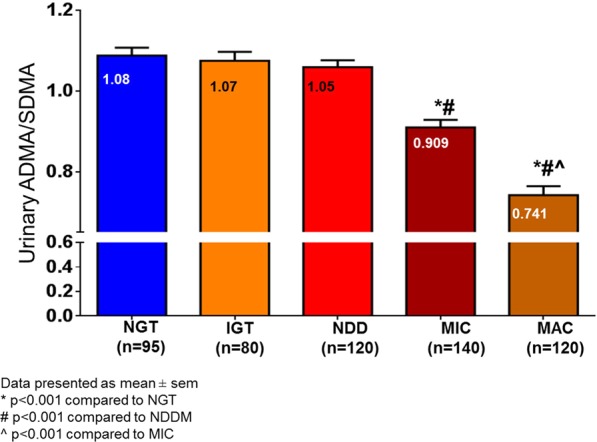
High-throughput analysis of urinary biomarker ADMA/SDMA ratio using MALDI-MS/MS.

ASR was measured using MALDI-MS/MS in the 555 urine samples across all the sample groups. ASR was significantly lower in MIC (0.909; p < 0.01) and MAC (0.741; p < 0.01) in comparison to the NGT and NDD groups while there were no significant differences between NGT, IGT and NDD groups (Fig. 1). To examine the influence of gender on the association of ASR with MIC and MAC, study subjects were stratified by gender. There were no significant differences in the ASR by gender in individuals with NDD, MIC, and MAC [Data not shown]. The clinical and biochemical characteristics of the study participants are shown in Table 1. Age, waist circumference, systolic and diastolic blood pressure, total cholesterol and serum triglycerides were significantly higher in patients with MIC (p < 0.05) and MAC (p < 0.05) compared to NDD, IGT, and NGT. Serum creatinine (p < 0.05) was higher and eGFR (p < 0.05) was lower in patients with MIC and MAC compared to NDD or NGT.

**Table 1:** Clinical and biochemical characterization of study subjects.

Variables	NGT(n = 95)	IGT(n = 80)	NDD (n = 120)	MIC (n = 140)	MAC (n = 120)
Male/Female	49/46	34/46	68/52	96/44	84/36
Age (years)	44 ± 10	48 ± 9.3	47 ± 10	54 ± 10 *#	56 ± 11*#
Duration of Diabetes (years)	—	—	—	13.1 ± 5.8	18.1 ± 7.2
Waist circumference (cm)	90 ± 11.0	95 ± 11.0*	96 ± 10*	96 ± 11*	98 ± 10*
Body mass index (kg/m^2^)	27 ± 4.6	29 ± 7.0	28 ± 4.1	27 ± 4.3	27 ± 4.5
Systolic blood pressure (mmHg)	124 ± 16	125 ± 15	124 ± 16	136 ± 19*#	139 ± 18*#
Diastolic blood pressure (mmHg)	79 ± 11	79 ± 9.0	80 ± 9.0	82 ± 9.0*	81 ± 12^
Fasting plasma glucose (mg/dl)	90 ± 13	107 ± 23	175 ± 63*	179 ± 77*	182 ± 85*
HbA1c (mmol/mol)	38 ± 2.7	43 ± 4.2	68 ± 17.8*	73 ± 18.2*	70 ± 17.9*
Total cholesterol (mg/dl)	178 ± 31	187 ± 37	197 ± 46*	169 ± 45#	173 ± 53#
Serum triglycerides (mg/dl)	127 ± 59	145 ± 85	169 ± 116	186 ± 115*	217 ± 188*#
Serum HDL cholesterol (mg/dl)	42 ± 9.3	42 ± 9.1	39 ± 9.0	37 ± 7.6*	38 ± 8.8*
Serum LDL cholesterol (mg/dl)	112 ± 25	118 ± 34	126 ± 42	96 ± 35 *#	94 ± 40 *#
Blood urea (mg/dl)	21 ± 5.2	21 ± 5.1	22 ± 6.1	27 ± 11*#	40 ± 23*^#^^
Serum creatinine (mg/dl)	0.72 ± 0.17	0.73 ± 0.16	0.76 ± 0.17	0.98 ± 0.33 *	1.5 ± 1.0*^#^^
eGFR (mL/min/1.73 m^2^)	107 ± 33	102 ± 23	102 ± 21	92 ± 31*^#^	63 ± 32*^#^^
Hypertension n (%)	8 (8.4)	21 (26.3)	37 (30.8)	81 (57.9)	88 (73.3)
Retinopathy n (%)	0	1 (1.3)	4 (3.3)	34 (24.3)	34 (28.3)
CAD n (%)	0	1 (1.3)	5 (4.2)	7 (5.0)	13 (10.8)
Insulin + OHA n (%)	0	0	0	82 (58.6)	99 (82.5)
OHA n (%)	0	0	0	58 (41.4)	21 (17.5)
Antihypertensive drug n (%)	6 (6.3)	16 (20.0)	32 (26.7)	75 (53.6)	81 (67.5)
Aspirin n (%)	0	0	5 (4.2)	10 (7.1)	16 (13.3)
Fenofibrate n (%)	7 (7.4)	11 (13.8)	26 (21.7)	34 (24.3)	27 (17.5)
Statin n (%)	16 (16.5)	28 (35.0)	52 (43.3)	89 (63.6)	77 (64.2)

*p < 0.05 compared to NGT; ^#^*p* < 0.05 compared to NDD; ^^^*p* < 0.05 compared to MIC.

ASR was negatively correlated with age (p < 0.001), systolic blood pressure (p < 0.001), fasting plasma glucose (p = 0.004) and glycated hemoglobin (p < 0.001), total cholesterol (p < 0.01), blood urea (p < 0.001), serum creatinine (p < 0.001) and MIC (p < 0.001). ASR showed a positive correlation with HDL cholesterol (p < 0.001) and eGFR (p < 0.001) (Supplemental Table 4).

Standardized polytomous logistic regression analysis was performed with NDD as the dependent variable and ASR as the independent variable (Table 2**)**. Every one standard deviation decrease in ASR was independently associated MIC [odds ratio (OR): 0.307, 95% confidence interval (CI): 0.213–0.444; p < 0.01]. This association remained statistically significant even after adjusting for age, gender, blood pressure, glycated hemoglobin, serum cholesterol, triglycerides, HDL and LDL cholesterol, urea, serum creatinine, eGFR, and duration of diabetes [OR: 0.256; 95% CI: 0.158–0.491; p < 0.01]. ASR was independently associated with MAC [OR per standard deviation: 0.138; 95% CI: 0.090–0.211; p < 0.01]. Adjustment for age, gender, blood pressure, glycated hemoglobin, serum cholesterol, triglycerides, HDL and LDL cholesterol, urea, serum creatinine, eGFR and duration of diabetes did not substantially change the association between ASR and MAC [OR per standard deviation: 0.146; 95% CI: 0.071–0.292; p < 0.01] **(**Table 2**)**.

**Table 2:** Standardized polytomous logistic regression estimates for the risk of MIC and MAC using NDD as reference.

VARIABLES	NDD Ref.	MIC OR (CI)	MAC OR (CI)
ADMA/SDMA Ratio – Independent variable*			
ADMA/SDMA ratio unadjusted	^1^	0.307 (0.213–0.444)*	0.138 (0.090–0.211)*
Model 1: Adjusted for age and gender	^1^	0.316 (0.213–0.468)*	0.147 (0.094–0.230)*
Model 2: Model 1 plus systolic, diastolic blood pressure and glycated hemoglobin		0.304 (0.196–0.473)*	0.145 (0.088–0.240)*
Model 3: Model 2 plus serum cholesterol, serum triglycerides, serum HDL and LDL cholesterol		0.287 (0.171–0.483)*	0.117 (0.064–0.215)*
Model 4: Model 3 plus blood urea, serum creatinine, eGFR and duration of diabetes		0.256 (0.158–0.491)*	0.146 (0.071–0.292)*

*p < 0.01 compared to NDD.

*Per standard deviation changes in ADMA/SDMA.

**Figure 2:**
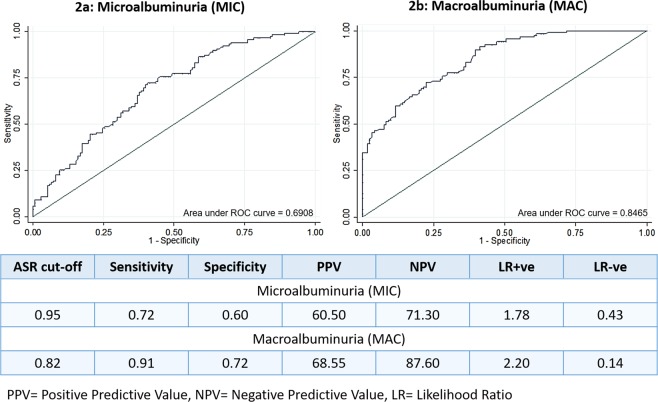
ROC curves of ASR among individuals with MIC and MAC.

Receiver operating characteristic curves (ROC) were constructed to derive the cut-point for ASR with the best sensitivity and specificity to identify MIC and MAC. Figure 2 shows the C-statistic for the ASR in predicting MIC and MAC. An ASR cut-point of 0.95 had a C-statistic of 0.691 (95% CI: 0.627-0.755; p < 0.001) indicating the high ability of ASR to discriminate MIC from NDD. The sensitivity and specificity of this ASR cut-off of ≥0.95 were 72% and 60%, respectively, for identifying patients with MIC. The positive predictive value was 60.5%, and the negative predictive value, 71.3%. An ASR cut-point of 0.82 had C statistic of 0.846 (95% CI: 0.800 - 0.893, p < 0.001) had a sensitivity of 91% and specificity of 72%, for identifying MAC. The positive predictive and the negative predictive values were 68.5% and 87.6% respectively.

## Discussion

Manifestation of albuminuria, overt nephropathy, and an eventual declining glomerular filtration rate (GFR) marks renal insufficiency and characterize progression of DN^33^. We earlier reported that the prevalence of overt nephropathy and MIC in urban Asian Indians was 2.2 and 26.9%, respectively^28^. Studies have shown that MIC is a poor predictor of DN^34–37^. On the other hand, MAC is a strong predictor of disease progression that only develops at an advanced stage of DN^35,38^. However, at this stage, little can be done to prevent the development of end-stage kidney failure. Therefore, early diagnosis using a sensitive biomarker is beneficial to detect the onset of DN and to prevent/delay the progression into overt nephropathy. In this context, our study using a novel and validated MALDI MS/MS approach reports the following significant findings: (1) ASR is lower in patients with MIC and MAC compared to NGT or NDD. (2) ASR shows a significant inverse correlation with age, systolic blood pressure, blood urea, serum creatinine, and MIC and positively correlated with HDL cholesterol and eGFR. (3) ASR is independently associated with higher risk of MIC and MAC even after adjusting for other risk variables known to be associated with nephropathy. (4) ASR cut-points of 0.95 and 0.82 had the best sensitivity and specificity for identifying MIC and MAC among Asian Indians respectively. Furthermore, while the ASR cut-points of 0.95 and 0.82 may be useful in South Asians, these cut points may differ in other ethnic groups. This study being cross-sectional in nature, no cause and effect relationship or mechanistic conclusions between ASR and NDD, MIC or MAC could be established.

ADMA and SDMA are important disease markers implicated in clinical findings across a spectrum of diseases that include cardiovascular, renal disorders, hypertension and diabetes^39–42^. ADMA and SDMA, having a nominal mass of 203 (C_8_H_18_N_4_O_2_), are generated from protein methylation biosynthetic pathway subsequent to hydrolysis. Both the isomeric DMAs have been previously investigated and reported as biomarkers for kidney disease^43^. Unlike ADMA, SDMA is largely excreted through urine and thus is strongly associated with renal function^44^. Urinary levels of ADMA and SDMA reflect the overall metabolism and might be more reliable markers of pathological states.

DMAs are susceptible to protein binding, which may result in differential recovery for normal and proteinuric urine samples. Therefore, any quantitative estimation of DMA isomer(s) and eventually the comparisons with controls would have to take this into account as urine from patients with micro and macro-albuminuria has significant protein content. However, both the isomers exhibit almost similar binding to proteins^45^ and hence estimation of their ratio offers a much better and robust alternative to individual quantitative measurements. Additionally, the measurement of ASR would also remain unaffected by hydration levels and urine output of the patient. Estimation of the ASR, measured using MALDI MS/MS in this study, overcomes these inherent challenges of metabolite variations associated with urine samples^31,46^ and allows for comparisons to be made across normal and proteinuric urine samples. Urinary ADMA to SDMA distribution has been earlier detected from various disease conditions^15–17,20^. In this context, the decreased ASR in patients with MIC and MAC in our study is an important finding. Additionally, our data suggest that an ASR cut-point of 0.95 and 0.82 could be used to correctly identify 72% of MIC and 91% of MAC respectively in this Asian Indian population.

Clinical studies have demonstrated an association of ADMA and SDMA levels with decreasing renal function in CKD after stratification of subjects according to GFR^47,48^. These studies included both diabetic and non-diabetic CKD subjects. The prognostic value of ADMA in patients with CKD was investigated by Fliser *et al*.^48^. They established that the ADMA levels in plasma was an independent predictor of progression of renal disease. These findings were corroborated by Ravani *et al*.^49^ who found ADMA to be independently associated with progression to dialysis. Duranton *et. al*. have investigated the independent profiles of plasma and urinary ADMA and SDMA in CKD^50^. These studies affirm the potential value of ADMA and SDMA for the prediction of severity of renal disease. In this context, our study finds a negative correlation between ASR and glycemic parameters that include glycated hemoglobin (HbA1c) and fasting glucose levels. The inverse correlation between ASR with serum creatinine and MIC suggests its potential diagnostic as well as prognostic value. Significantly, the positive correlation of ASR with eGFR also substantiates the role of ASR in assessing diabetic kidney injury. Interestingly, the association of ASR with MIC or MAC is significant even after adjusting the conventional risk factors for DN and emphasizes a potential risk factor role of ASR. Decreased ASR may, therefore, represent a biomarker for MIC and MAC. However, this needs to be confirmed by longitudinal studies. Our work is a proof-of-concept that shows the use of ASR for assessing progressive DN.

To our knowledge, this is the first report that measures the urinary DMA distribution from different stages of diabetes. The strength of the study is that the cases (MIC, MAC/NDD) and IGT and controls (NGT), classified using standard methods, are statistically significant. Direct estimation of the ASR overcomes the inherent challenges of metabolite variations in urine samples and allows for comparisons to be made across disease conditions.

In summary, we report that the ASR profile is lower in MIC and MAC suggesting that it has the potential to be used as an early diagnostic marker. ASR cut-points of 0.95 and 0.82 could be used to correctly identify 72% of MIC and 91% of MAC respectively among Asian Indians, a population that is currently considered the epicenter of worldwide diabetes epidemic. Prospective studies are needed to further understand the mechanisms that govern decreased ASR and the factors that could be beneficially used to neutralize their cellular action. This would be invaluable both from a prevention as well as for novel therapeutic perspective towards better management of DN.

## Supplementary information

Supplementary Information
