# Supplementary material for: Plausible diagnostic value of urinary isomeric dimethylarginine ratio for diabetic nephropathy
Source: Sci Rep. 2020 Feb 19;10:2970. doi: 10.1038/s41598-020-59897-1 (PMC7031402; doi:10.1038/s41598-020-59897-1)
Supplement: Supplementary file 1 — Supplementary Information [file 41598_2020_59897_MOESM1_ESM.doc]

**Supplemental File**

**Plausible diagnostic value of urinary isomeric dimethylarginine ratio for diabetic nephropathy**

Dharmeshkumar Parmar^1,4,#^, Nivedita Bhattacharya^1,4,#^, Shanthini Kannan^1,2^, Sangeetha Vadivel^1,2^, Gautam Kumar Pandey^2^, Avinash Ghanate^1,4^, Nagarjuna Chary Ragi^5^, Paramasivam Prabu^2^, Thyparambil Aravindakshan Pramodkumar^2^, Nagaraj Manickam^3^, Viswanathan Mohan^2^, Prabhakar Sripadi^4,5^, Kuppan Gokulakrishnan^2,6*^ & Venkateswarlu Panchagnula^1,4*^

^1^Biochemical Engineering, CSIR-National Chemical Laboratory, Dr. Homi Bhabha Road, Pune India – 411008

^2^Department of Research Biochemistry, Madras Diabetes Research Foundation, No. 4, Conran Smith Road, Gopalapuram, Chennai India - 600086

^3^Department of Vascular Biology, Madras Diabetes Research Foundation, No. 4, Conran Smith Road, Gopalapuram, Chennai India - 600086

^4^Academy of Scientific and Innovative Research, CSIR-NCL Campus, Dr. Homi Bhabha Road, Pune India – 411008

^5^CSIR-Indian Institute of Chemical Technology, Uppal Road, Hyderabad India - 500007

^6^Department of Neurochemistry, National Institute of Mental Health and Neurosciences, Hosur Road, Bengaluru, India - 560029

**Key-words:** **Key-words:** Diabetes, Urine, Microalbuminuria, Macroalbuminuria, Dimethyl arginine, ADMA/SDMA, high-throughput, MALDI- MS/MS

^#^Contributed equally

^*^Corresponding authors

VP Phone: +91 20 2590 2194; Fax: +91 20 2590 2621; Email: [v.panchagnula@ncl.res.in](mailto:v.panchagnula@ncl.res.in);

KG Phone: +91 996 246 5824; Email: [gokulmdrf@gmail.com](mailto:gokulmdrf@gmail.com)

**Anthropometric and Biochemical measurements**

Anthropometric measurements including weight, height, and waist circumference, were obtained by trained data collectors using standardized methods ^1^. Body mass index (BMI) was calculated as weight (kg)/height (m)^2^. Blood pressure was recorded from the right arm in a sitting position to the nearest 2 mmHg with a mercury sphygmomanometer (Diamond Deluxe BP apparatus, Pune, India). Two readings were taken 5 minutes apart and the mean of the two was taken as the blood pressure.

Fasting plasma glucose (hexokinase method), serum cholesterol (cholesterol oxidase-peroxidase-amidopyrine method), serum triglycerides (glycerol phosphate oxidase-peroxidase-amidopyrine method) and HDL cholesterol (direct method-polyethylene glycol-pretreated enzymes), were measured using Hitachi-912 Autoanalyser (Hitachi, Mannheim, Germany). Low-density lipoprotein (LDL) cholesterol was calculated using the Friedewald formula. Glycated haemoglobin (HbA1c) was measured by high-pressure liquid chromatography using the Variant machine (Bio-Rad, Hercules, Calif., USA). Serum insulin concentration was estimated using the electrochemiluminescence method (COBAS E 411; Roche Diagnostics). Blood urea and serum creatinine (CRE) (Jaffe's method) were measured using a Hitachi-912 Autoanalyser (Hitachi, Mannheim, Germany). The intra- and inter-assay coefficients of variation for the biochemical assays ranged between 3.1% and 7.6%.

Urinary albumin was measured in fasting urine sample using immunoturbidimetric assay (Hitachi 902 autoanalyzer; Roche Diagnostics). eGFR was calculated by using CKD-epi equation. The CKD-EPI equation was calculated as GFR (ml/min/1.73 m2) = 141× min (serum creatinine/k, 1)α ×max (serum creatinine/k, 1)−1.209 ×0.993Age ×1.018 (if women) × 1.159 (if black), where k is 0.7 for women and 0.9 for men, α is −0.329 for women and −0.411 for men; ‘min’ indicates minimum serum creatinine/k or 1, and ‘max’ indicates maximum serum creatinine/k or 1 ^2^. All measurements were performed in a laboratory that is certified by the College of American Pathologists (Northfield, IL) and the National Accreditation Board for Testing and Calibration of Laboratories (New Delhi, India).

**Definitions:**

Diabetes was defined using World Health Organization consulting group criteria ^3^. Those who were confirmed by oral glucose tolerance test to have fasting plasma glucose ≥7.0 mmol/L (≥126 mg/dL) and/or 2-hour plasma glucose value ≥11.1 mmol/L (≥200 mg/dL) or past medical history (self-reported diabetes under treatment by a physician), or drug treatment for diabetes (insulin or oral hypoglycemic agents) were diagnosed as T2DM. Those with fasting plasma glucose <5.6 mmol/L (<100 mg/dl) and 2-hour post glucose value ≥7.8 mmol/L (≥140 mg/dL) and <11.1 mmol/L (<200 mg/dL) were diagnosed as IGT, and those with fasting plasma glucose <5.6 mmol/L (<100 mg/dl) and 2-hour post glucose value <7.8 mmol/L (<140 mg/dL) as NGT ^3^. MIC was diagnosed if the albumin excretion was between 30 and 299 μg/mg of creatinine. The diagnostic criterion for MAC or overt nephropathy was albumin excretion exceeding 300 μg/mg of creatinine ^4^. Regarding the selection of subjects, we have carefully followed them for their previous visits and only those subjects with three consecutive values of 30–299 μg/mg creatinine were considered micro-albuminuric and ≥300 μg/mg creatinine were considered to be macro-albuminuric patients.

**Statistical analysis:**

We compared anthropometric, clinical, and biochemical characteristics of groups using one-way ANOVA [with Tukey’s HSD] for continuous variables and the Chi-square test or Fisher’s exact test to compare proportions. We performed Pearson correlation analysis to examine the correlation of various risk factors with ASR ^5^. Standardized polytomous regression analysis was done to assess how incremental changes in ASR were associated with MIC and MAC. We adjusted models for any demographic, anthropometric, clinical, or biochemical characteristics that were significantly different across groups ^6^.

Receiver operating characteristic curves (ROC) were plotted for ASR to identify MIC and MAC. Sensitivity, specificity, positive and negative predictive values, and accuracy for predicting MIC and MAC were calculated for various ASR cut points. C statistic or the area under the ROC (AROC) was estimated and by interpolation from the area under the curve, the point closest to the upper-left corner, which maximized sensitivity and specificity, was selected as the optimal cut point; this identified the highest number of subjects with or without MIC or MAC ^7^. All analyses were done using Windows-based SPSS statistical package (version 12.0, Chicago, IL).

## LC-MS/MS and MALDI MS/MS analysis

24 processed samples, 5 each from NGT, IGT, NDD, MIC and 4 from MAC were used for cross-platform validation on an Agilent 6420 triple quadrupole mass spectrometer with electrospray ionization (ESI) interface. For LC-MS/MS analysis, the samples were reconstituted in 160 µl of acetonitrile: water (50:50) and maintained at 4˚C in the autosampler. Injection volume was set to 5µL. The separation was achieved using Agilent ZORBAX HILIC PLUS column (100mm*4.6mm*3.5µm) with an isocratic mobile phase consisting of acetonitrile : water (90:10) with 0.3% acetic acid and 0.0125% trifluoroacetic acid, flow rate 600 µL/min, and a total run time of 9 min. The column temperature was maintained at 28±2.5 ˚C. The ESI capillary temperature, sheath gas and auxiliary gas were set at 300˚C, 45, and 15 arbitrary units, respectively. The mass spectrometer was operated in positive ion mode with ionization voltage 4000 V. Ion transitions, m/z 203.1→46.1 for ADMA and m/z 203.1→172.1 for SDMA, were monitored in multiple reaction monitoring modes (MRM)^8,9^. MS/MS for ADMA and SDMA was performed at 20 eV, 13 eV, respectively. Data analysis was carried out by the MassHunter software, version B.07.00.

**Supplemental Figure 1** MALDI-MS/MS spectra of urinary isomeric dimethylarginine (m/z 203) from (a) normoglycemic (NGT) and (b) type 2 diabetes with macroalbuminuria (MAC) samples. The diagnostic product ions at *m/z* 46 (<10ppm error) and *m/z* 172 (<5ppm error) represent ADMA and SDMA, respectively. S/N (>100) for both *m/z* 46 and 172 were obtained in all the samples.


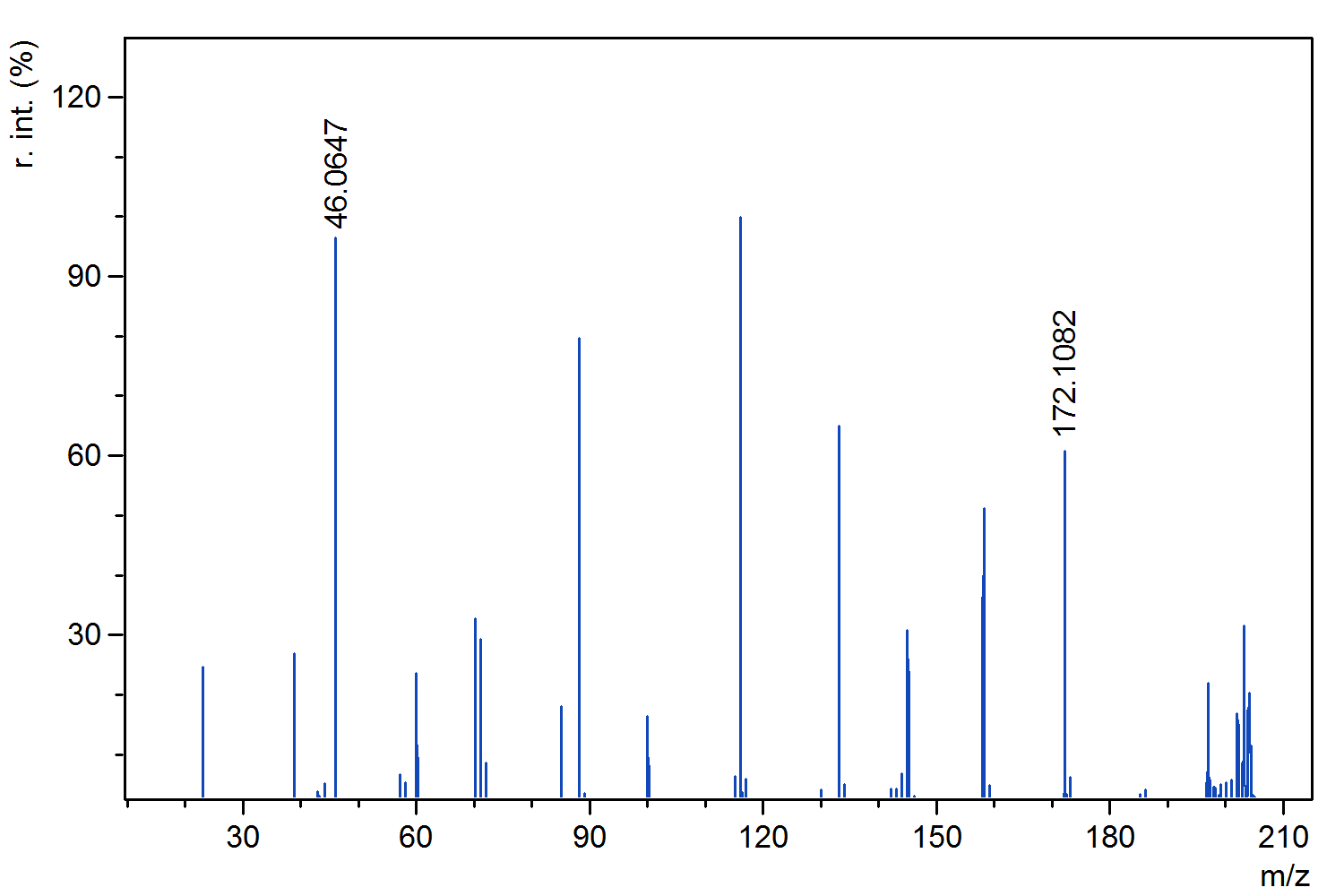

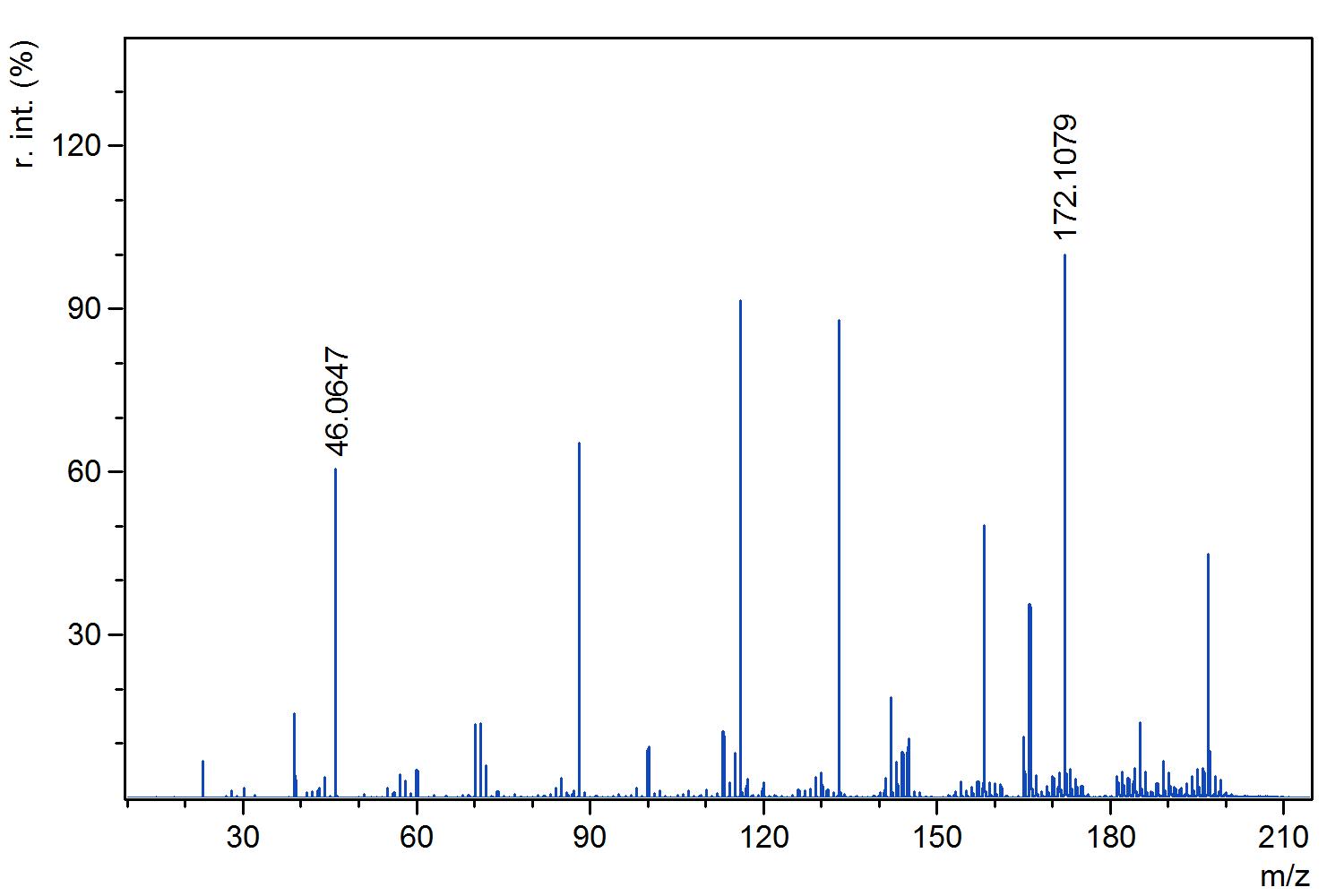


(a)

(b)

**Supplemental figure 2 Schematic representation of analytical workflow for method validation**

**Supplemental figure 2** Schematic representation of analytical workflow for method validation


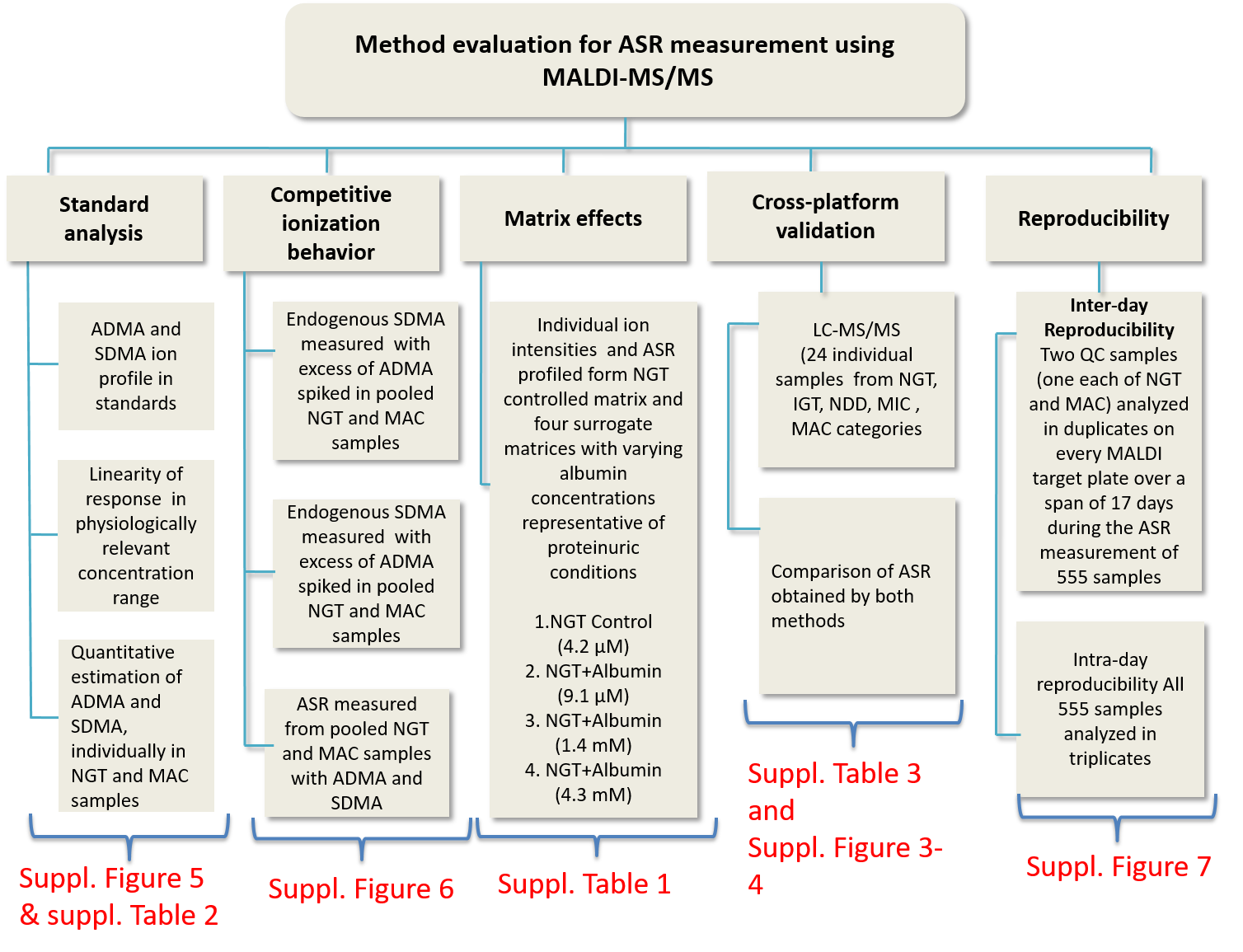


**Supplemental figure 3** Correlation plot (n=22) of urinary ASR measured using MALDI-MS/MS and LC-MS/MS


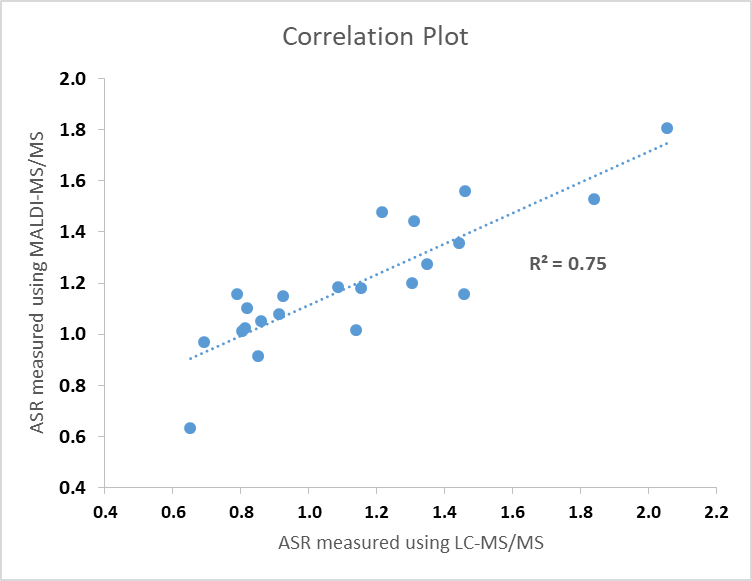


**Supplemental Figure 4** A representative extracted ion chromatogram of ADMA (*m/z* 203 🡪 46) and SDMA (*m/z* 203 🡪 172) from an IGT sample acquired using LC-MS/MS.


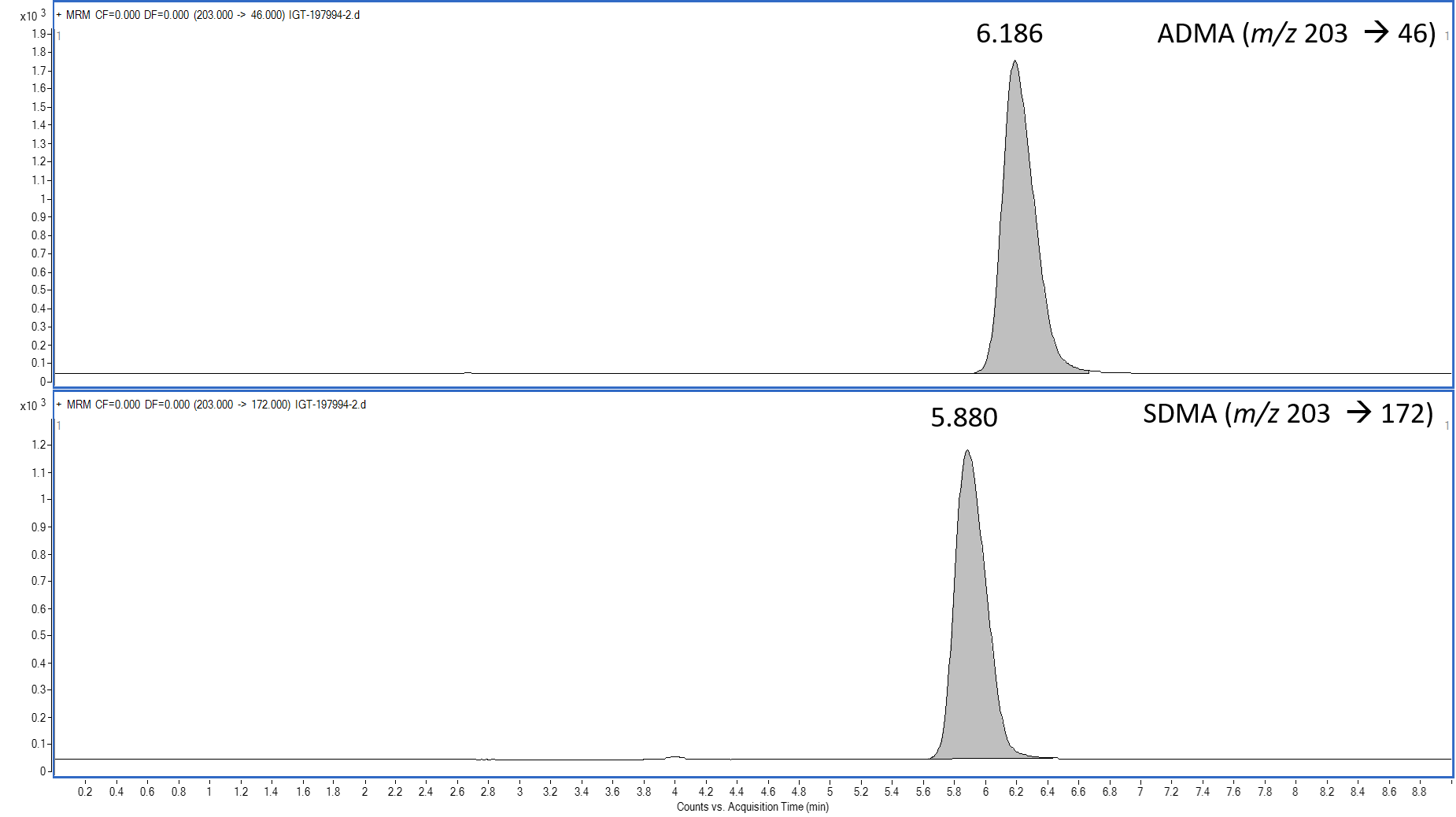


**Supplemental Figure 5** MALDI-TOF MS/MS peak intensity profiles of ADMA (*m/z* 46) and SDMA (*m/z* 172) as measured in standard solution (a) are shown. Three concentration levels of ADMA and SDMA (1:1) were used – 9 µM, 30 µM and 45 µM and a proportional increase in the intensity for the respective unique product ions was observed. The peak intensities of ADMA and SDMA were overlapping at each of the spiked levels. Sample matrix-associated ion suppression effects, variability owing to the presence of protein (in MIC and MAC urine samples), inter and intra assay reproducibility were further investigated. ADMA and SDMA standards were also spiked in pooled NGT (b) and MAC (c) samples with similar trends observed. The corresponding *m/z* 46/172 ASR estimated as a ratio of peak intensities (from a, b & c) is relatively uninfluenced by the three concentration levels (d). These results indicate that the MS/MS ionization efficiencies were similar for both the isomers, responses were linear and the urine matrix-associated ion suppression effects (represented by two extremes of the urine samples NGT and MAC) were negligible.

**
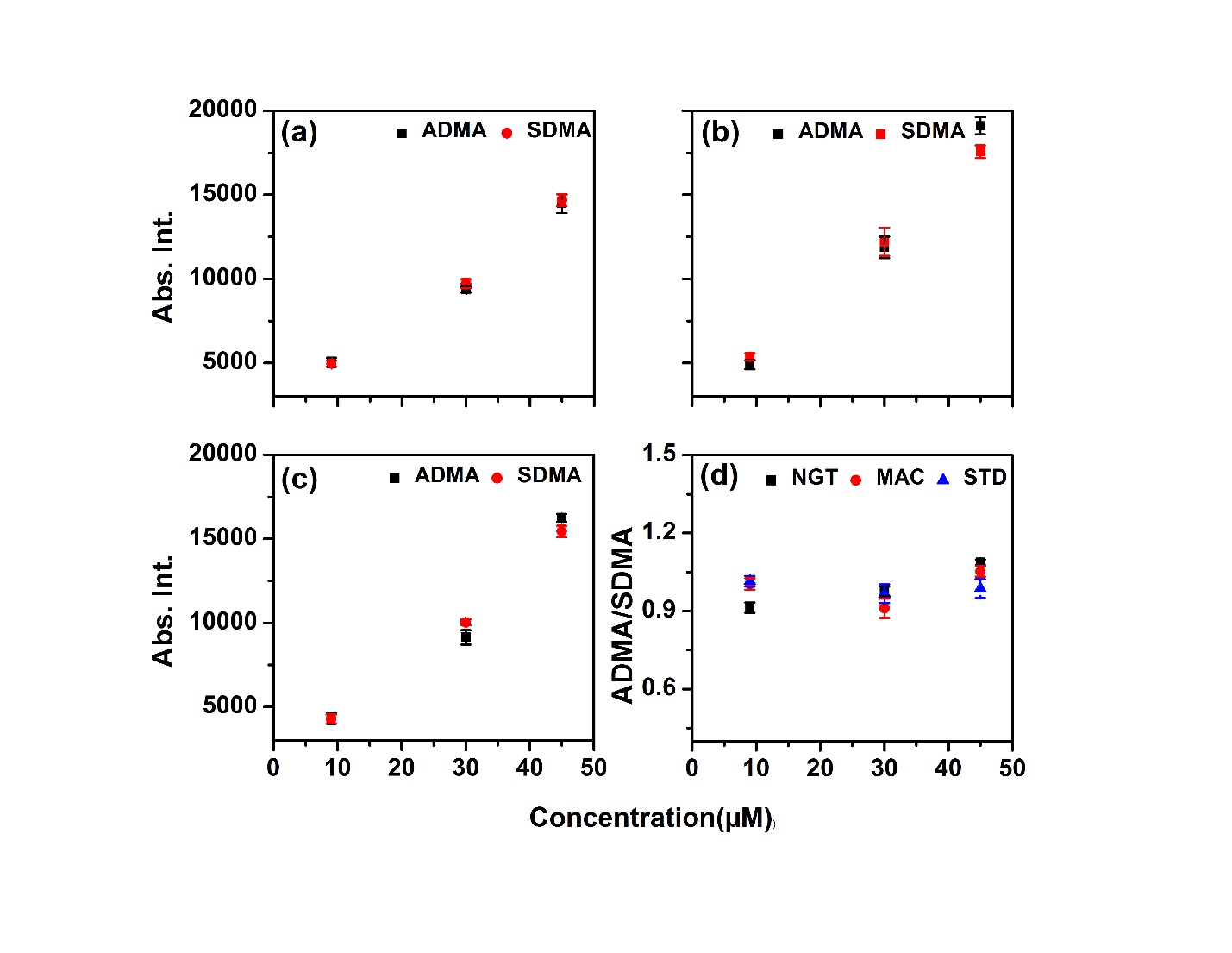
**

**Supplemental Figure 6** Low abundant analytes could potentially encounter inefficient ionization and resultant signal suppression in the presence of highly abundant analytes of similar m/z and chemical nature. To rule out any such competitive ionization between ADMA and SDMA, the effect of excess analyte (spiked) on the endogenous (non-spiked) counterpart was investigated. In the first case, three concentration levels of SDMA were spiked in the NGT and MAC samples (in significant excess, 18 µM, 60 µM and 90 µM in the final solution) and the endogenous ADMA levels were monitored using MALDI TOF MS/MS (a). Marginal increase in the absolute peak intensities were observed for ADMA in the MAC pooled sample in the presence of 60 and 90 µM spiked SDMA. No significant peak intensity change was observed for SDMA in MAC and NGT samples. Likewise, SDMA measured in the presence of excess spiked ADMA at the three levels (18 µM, 60 µM and 90 µM) showed no significant enhancement or suppression in both NGT and MAC samples. All the peak intensities were observed reproducibly with low standard deviations as indicated by the error bars. The outcomes of these investigations indicate an absence of abundant analyte associated competitive or selective ionization of ADMA and SDMA from NGT and MAC samples. Thus, any variations of ASR measured from urine samples would in all likelihood be free from ion suppression effects and uninfluenced by relative abundancies of the respective metabolites.


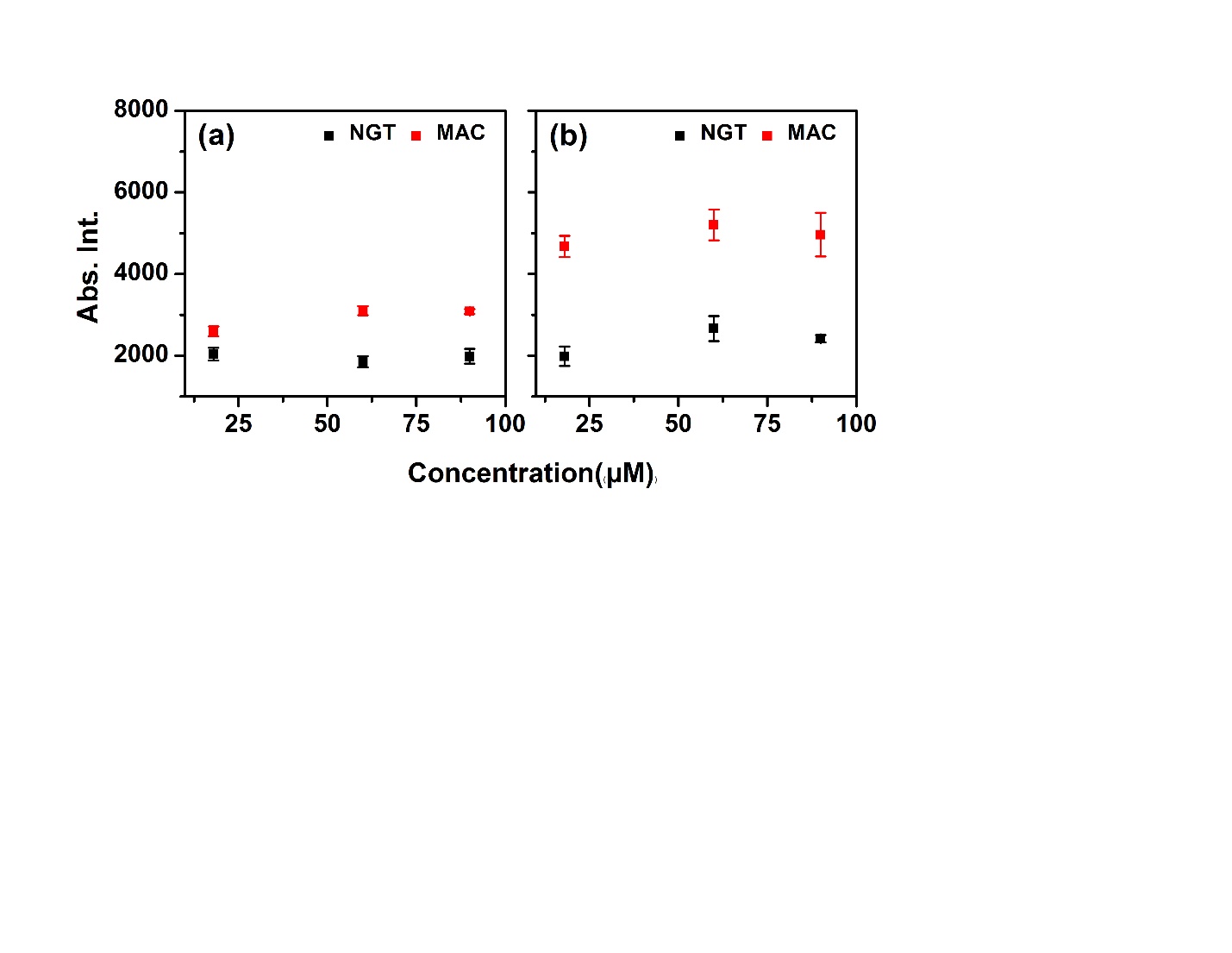


**Supplemental Figure 7** Reproducibility of MALDI-MS/MS measurement, for the span of sample data acquisition, was evaluated by monitoring QC control samples. Box-Whiskers plot representing inter-day reproducibility of two quality control (QC1 and QC2) samples used over the course of MALDI-TOF MS/MS analysis of 555 clinical samples. The data was acquired in duplicates on each occasion and compiled for 18 MALDI target plates (analyzed over several days; 36 data points for each QC). A standard deviation (SD) of 0.075 (average ASR=0.85) and 0.063 (average ASR=0.39) were observed for QC1 and QC2, respectively. The data encompassing 18 MALDI target plates indicate that the ASR can be reproducibly measured in a clinical sample using MALDI-MS/MS over a period of several days and the data comparisons can be drawn. Any prolonged use of MALDI-MS/MS for the measurement of ASR in a study of larger sample set may require the use of QC checks and monitoring of reproducibility to ensure any time-associated variations.


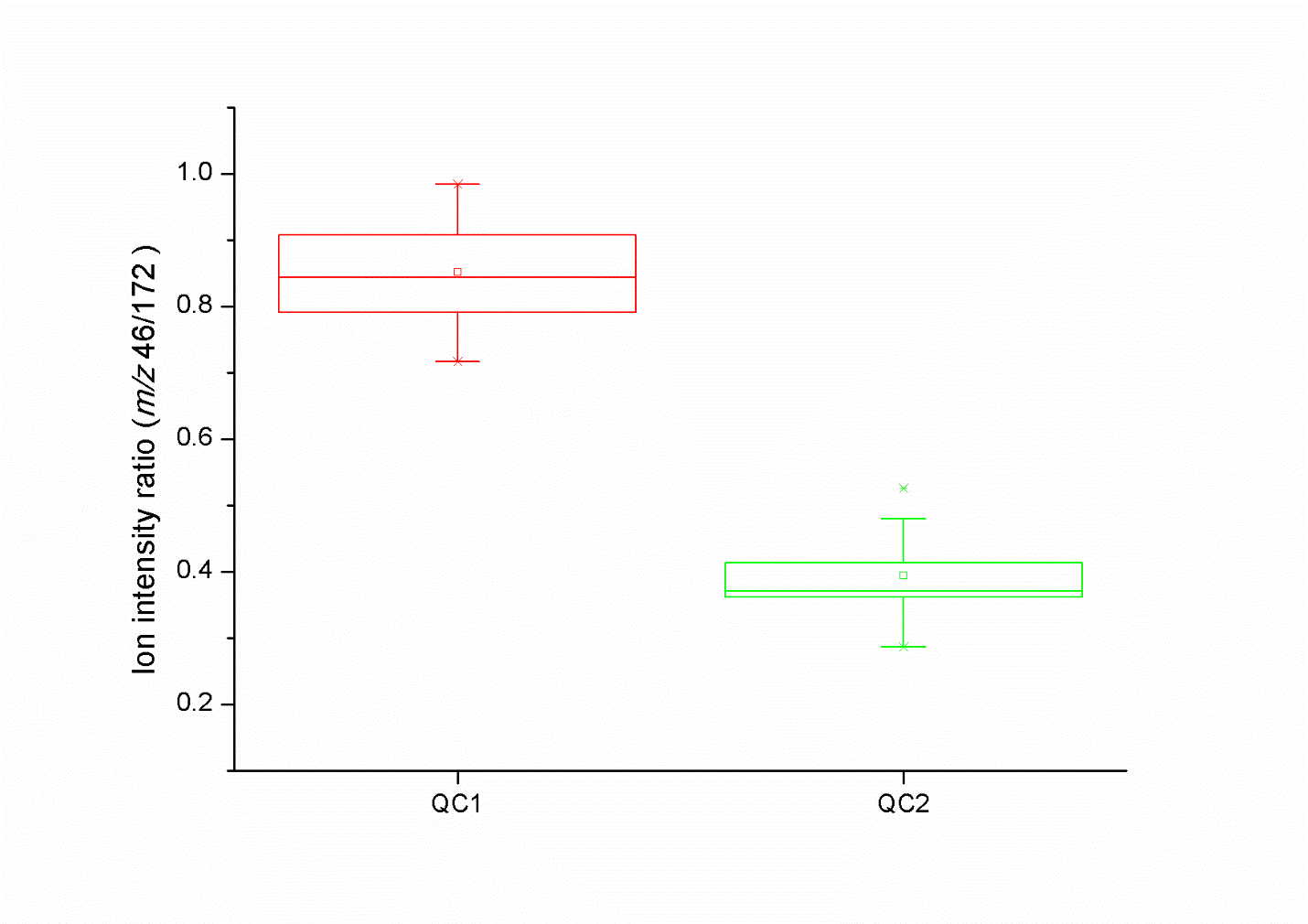


**Supplemental Table 1.** Influence of protein content on ASR measurement: ASR was measured in NGT (control) and albumin spiked NGT samples (spiked NGT* 1 to 4).

| **Sample Name** | **Replicate** | ***m/z* 46** | ***m/z* 172** | ***m/z* 46/172** | **Replicate avg. (%RSD)** | **% Variation from control** |
| --- | --- | --- | --- | --- | --- | --- |
| NGT* (control) | **R1** | 7.53E+03 | 6.19E+03 | 1.22 | 1.20 (4.7) | 00.00 |
|  | **R2** | 4.97E+03 | 3.97E+03 | 1.25 |  |  |
|  | **R3** | 5.16E+03 | 4.53E+03 | 1.14 |  |  |
| Spiked NGT* 1  (4.2 µM of albumin ) | **R1** | 1.73E+03 | 1.52E+03 | 1.13 | 1.11(5.30) | -7.09 |
|  | **R2** | 1.80E+03 | 1.71E+03 | 1.05 |  |  |
|  | **R3** | 1.32E+03 | 1.14E+03 | 1.16 |  |  |
| Spiked NGT* 2  (9.1 µM of albumin ) | **R1** | 1.13E+03 | 9.28E+02 | 1.22 | 1.13 (7.51) | -6.19 |
|  | **R2** | 1.07E+03 | 9.74E+02 | 1.10 |  |  |
|  | **R3** | 1.04E+03 | 9.78E+02 | 1.06 |  |  |
| Spiked NGT* 3 (1.4 mM albumin) | **R1** | 2.67E+03 | 2.54E+03 | 1.05 | 1.08 (2.8) | -10.00 |
|  | **R2** | 2.74E+03 | 2.47E+03 | 1.11 |  |  |
|  | **R3** | 2.36E+03 | 2.19E+03 | 1.08 |  |  |
| Spiked NGT* 4 (4.3 mM albumin) | **R1** | 5.70E+03 | 4.71E+03 | 1.21 | 1.33 (8.7) | +10.83 |
|  | **R2** | 4.26E+03 | 3.20E+03 | 1.33 |  |  |
|  | **R3** | 3.19E+03 | 2.21E+03 | 1.44 |  |  |

NGT* = Pooled sample (n=5)

To understand the impact of higher protein content in micro and macro-albuminuria, the change in ASR was monitored in albumin spiked pooled NGT sample (*n*=5). The average normal concentration of albumin in human urine is 30 µg/mg of creatinine. This corresponds to an absolute concentration of albumin being 377.25 µM. As per the NKF-KDOQI (National Kidney Foundation–Kidney Disease Outcomes Quality Initiative) guidelines, the albumin excretion rate (AER) for MIC and MAC are 30-300 µg/mg and >300 µg/mg creatinine, respectively.^10,11^ Corresponding to the guidelines, pooled NGT samples were spiked with albumin to obtain a representative albumin concentration for MIC and MAC categories. To understand the influence of variable albumin concentration on the measurement of ASR, 100 µL pooled NGT urine was spiked with albumin to bring the albuminuria content level to MIC and MAC, respectively. The final concentration of albumin in the spiked sample was 4.263 µM (spiked NGT* 1), 9.1 µM (spiked NGT* 2), 1.4 mM (spiked NGT* 3), and 4.3 mM (spiked NGT* 4). Spiked sample NGT* 1 and NGT* 2 represented albumin level within the range of MIC and MAC samples. Supplemental table 2 contains the details of replicate data for control and albumin spiked NGT* samples. The RSD of technical replicates was found to be <10 % RSD, indicating acceptable reproducibility. The changes in the ASR in control and spiked samples showed <10% variation from the control sample indicating the negligible influence of albumin content on ASR measurement using MALDI-MS/MS. Spiked sample NGT* 3 and NGT* 4 were prepared in extreme excess albumin content (~1000 times) to further evaluate the influence of albumin on ASR measurement. The albumin content in spiked sample NGT* 3 and 4 is practically impossible in real life scenario. In spite of the extreme levels of albumin, the reproducibility of ASR measurement remained unaffected. The changes in the ASR in control and spiked samples showed ~10% variation from the control sample indicating the error threshold beyond which any changes could be associated with the disease condition.

**Supplemental Table 2.** Details of the ASR (ADMA/SDMA) estimated using absolute intensity and concentration. ADMA and SDMA were monitered through unique product ions m/z 46 and 172, respectively.

| **Analyte** | **Concentration in µM**  **(±standard Deviation)** | | |
| --- | --- | --- | --- |
|  | **ADMA** | **SDMA** | **ASR** |
| **NGT^a^** | 5.73(0.58) | 6.85(1.21) | 0.88(0.07) |
| **MAC^a^** | 1.18(0.05) | 2.82(0.01) | 0.42(0.02) |

^a^ Represents pooled sample as described in the methodology section. The individual urinary concentrations of each isomer was determined using respective standard calibration curves. The calibration range, R^2^ for the calibration equations, and the QC recoveries for both isomers were 0.06 – 9.00 µM, 0.99, and within ±20% of expected value respectively. The ASR values and overall trend determined using two approaches were found to be comparable (See supplemental figure 7 for comparison).

**Supplemental Table 3.** ASR (%RSD) values of 24 samples measured on MALDI MS/MS and LC-MS/MS (triplicates). The transitions m/z 203 to 46 and 203 to 172 indicative of the presence of ADMA and SDMA were monitored from the urine samples as well as reference standards.

| **Category** | **Sample No.** | **MALDI MS/MS** | **LC-MS/MS** |
| --- | --- | --- | --- |
| **NGT** | 1 | 1.077(4.9%) | 1.546(1.3%) |
|  | 2 | 1.196(12.2%) | 1.180(0.3%) |
|  | 3 | 1.144(1.9%) | 1.017(0.4%) |
|  | 4 | 1.427(8.0%) | 1.560(0.4%) |
|  | 5 | 1.332(5.5%) | 1.275(0.5%) |
| **IGT** | 1 | 1.305(2.5%) | 1.442(1.7%) |
|  | 2 | 1.842(5.4%) | 1.530(0.4%) |
|  | 3 | 1.086(1.2%) | 1.184(0.4%) |
|  | 4 | 1.372(5.2%) | 0.903(0.4%) |
|  | 5 | 0.818(1.7%) | 1.104(0.7%) |
| **NDD** | 1 | 1.216(5.6%) | 1.478(2.2%) |
|  | 2 | 2.055(8.3%) | 1.808(0.7%) |
|  | 3 | 1.444(14.5%) | 1.358(0.5%) |
|  | 4 | 0.924(6.0%) | 1.148(0.7%) |
|  | 5 | 1.306(9.6%) | 1.201(0.3%) |
| **MIC** | 1 | 1.457(5.1%) | 1.157(0.5%) |
|  | 2 | 0.915(5.3%) | 1.080(0.2%) |
|  | 3 | 0.723(2.8%) | 1.156(1.0%) |
|  | 4 | 0.825(2.1%) | 1.026(1.2%) |
|  | 5 | 0.852(2.0%) | 0.916(1.0%) |
| **MAC** | 1 | 0.720(3.1%) | 0.968(0.8%) |
|  | 2 | 0.841(3.0%) | 1.033(2.5%) |
|  | 3 | 0.805(7.7%) | 1.012(0.5%) |
|  | 4 | 0.470(15.2%) | 0.632(0.5%) |

**Supplemental Table 4.** Correlation analysis of ADMA/SDMA with metabolic risk factors

| **Parameter** | **ADMA/SDMA** | |
| --- | --- | --- |
|  | **r value** | **p value** |
| Age | **-0.267** | **p<0.001** |
| Body mass index | **0.047** | 0.278 |
| Waist circumference | **-0.091** | 0.056 |
| Systolic blood pressure | **-0.189** | **p<0.001** |
| Diastolic blood pressure | **0.001** | 0.981 |
| Fasting plasma glucose | **-0.136** | **p= 0.004** |
| Glycated hemoglobin | **-0.176** | **p<0.001** |
| Total cholesterol | **-0.165** | **p<0.01** |
| Serum triglycerides | **-0.077** | 0.088 |
| Serum HDL cholesterol | **0.161** | **p<0.001** |
| Serum LDL cholesterol | **-0.089** | 0.059 |
| Blood urea | **-0.323** | **p<0.001** |
| Serum creatinine | **-0.321** | **p<0.001** |
| eGFR | **0.334** | **p<0.001** |
| Microalbumin | **-0.519** | **p<0.001** |

**References**

1. Deepa, M. *et al.* The Chennai urban rural epidemiology study (CURES) - study design and methodology (Urban Component) (CURES - 1). *J. Assoc. Physicians India* **51**, 863–870 (2003).

2. Levey, A. S. *et al.* A new equation to estimate glomerular filtration rate. *Ann. Intern. Med.* **150**, 604–12 (2009).

3. Alberti, K. G. & Zimmet, P. Z. Definition, diagnosis and classification of diabetes mellitus and its complications. Part 1: diagnosis and classification of diabetes mellitus provisional report of a WHO consultation. *Diabet. Med.* **15**, 539–553 (1998).

4. Unnikrishnan, R. I. *et al.* Prevalence and risk factors of diabetic nephropathy in an urban South Indian population: the Chennai Urban Rural Epidemiology Study (CURES 45). *Diabetes Care* **30**, 2019–24 (2007).

5. Reddy, S. *et al.* Association of increased levels of MCP-1 and cathepsin-D in young onset type 2 diabetes patients (T2DM-Y) with severity of diabetic retinopathy. *J. Diabetes Complications* **31**, 804–809 (2017).

6. Gokulakrishnan, K. *et al.* Relationship of betatrophin with youth onset type 2 diabetes among Asian Indians. *Diabetes Res. Clin. Pract.* **109**, 71–76 (2015).

7. Hanley, A. J. & McNeil, J. B. The meaning and use of the area under a receiver operating characteristic (ROC) curve. *Radiology* **143**, 29–36 (1982).

8. Brown, C. M., Becker, J. O., Wise, P. M. & Hoofnagle, A. N. Simultaneous determination of 6 L-arginine metabolites in human and mouse plasma by using hydrophilic-interaction chromatography and electrospray tandem mass spectrometry. *Clin. Chem.* **57**, 701–709 (2011).

9. Paglia, G., D’Apolito, O., Tricarico, F., Garofalo, D. & Corso, G. Evaluation of mobile phase, ion pairing, and temperature influence on an HILIC-MS/MS method for L-arginine and its dimethylated derivatives detection. *J. Sep. Sci.* **31**, 2424–2429 (2008).

10. Inker, L. A. *et al.* KDOQI US commentary on the 2012 KDIGO clinical practice guideline for the evaluation and management of CKD. *Am. J. Kidney Dis.* **63**, 713–735 (2014).

11. Uhlig, K. & Eckardt, K.-U. A decade after the KDOQI CKD guidelines: impact on CKD guidelines. *Am J Kidney Dis* **60**, 705–6 (2012).
